# Effects of the Carbon Credit Policy on the Capital-Constrained Manufacturer’s Remanufacturing and Emissions Decisions

**DOI:** 10.3390/ijerph20054352

**Published:** 2023-02-28

**Authors:** Yongjian Wang, Fei Wang, Wenbo Li

**Affiliations:** 1 Business School, Jiangsu Normal University, Xuzhou 221116, China; 2 National Tax Institute of STA, Yangzhou 225007, China

**Keywords:** capital constraint, carbon credit policy, manufacturing/remanufacturing decisions, carbon threshold, preferential interest rate

## Abstract

Considering the effect of carbon emission factors on financing, a carbon credit policy was introduced to explore the capital-constrained manufacturer’s remanufacturing and carbon emission decisions. Meanwhile, this paper also explored the bank’s optimal strategy according to the manufacturer’s decision feedback. The results showed the following: (1) The restraining effect of the carbon threshold will directly affect whether the carbon credit policy can positively affect manufacturers’ remanufacturing and carbon emissions. (2) When the carbon savings level of remanufactured products is higher, the carbon credit policy can better promote remanufacturing activities and effectively control total carbon emissions. (3) The bank’s optimal preferential interest rate for loans is inversely correlated with the carbon threshold. Moreover, under a given carbon threshold, a higher preferential interest rate for loans is also conducive to manufacturers carrying out more or a more extensive range of remanufacturing activities while banks maximize total profit. Based on these findings, this paper also provided managerial insights for manufacturers and policy implications for policy-makers.

## 1. Introduction

The proposal of carbon peak and carbon neutral strategic goals indicates that China will face increasingly severe pressure to reduce carbon emissions. Remanufacturing is regarded as an essential means to reduce environmental pollution from manufacturing industries to restore the value of waste products and achieve emission reduction [1]. Studies and practices also show that remanufacturing activities have considerable economic and environmental benefits [2,3]. Therefore, more and more manufacturers have recognized and carried out remanufacturing activities, such as Canon, SANY, XGMA, and XCMG [4,5,6]. Taking XCMG Construction Machinery as an example, during 2015–2019, it achieved a remanufacturing output value of more than CNY 3.2 billion and reduced carbon dioxide emissions by about 266,000 tons by remanufacturing more than 1400 complete truck cranes and more than 11,000 core components [7]. For manufacturers, the product type will affect the total carbon emissions of the production operation process. Then, under the emissions constraint such as mandatory carbon threshold, the manufacturer’s remanufacturing and emissions decisions show a mutual influence coupling relationship.

However, due to the relatively high initial investment cost of remanufacturing activities and the relatively low market share of remanufactured products, many manufacturers encounter the problem of capital shortage [8,9]. Studies have also shown that one of the most important challenges that many manufacturers face in the process of low-carbon transformation is that it is difficult to obtain long-term financing support for green investment, especially for small- and medium-sized enterprises [10]. A survey of 2700 enterprises in China conducted by the World Bank Group reveals that 62% of enterprises are SMEs, and 54.5% need loans to meet their capital needs [11]. Therefore, relying solely on administrative restraints is impossible to improve the deteriorating environmental situation. It is also necessary to use financial regulatory instruments to curb the blind expansion of high-polluting and high-energy-consuming industries. In an open economy, the green transformation of an industry is increasingly influenced by the external environment, especially the performance of banks and the government [12]. To alleviate manufacturers’ financial difficulties and encourage them to engage in low-carbon production activities actively, many banks have launched a green credit business as a special financing way to support green innovation [13]. The carbon credit policy is a financial service provided by banks that integrates the low-carbon concept into the green credit policy to encourage enterprises to reduce carbon emissions. Carbon finance has also been proven to play an important role in the green transformation of industrial structure [14]. Under the carbon credit policy, when total carbon emissions reach the agreed thresholds, enterprises are entitled to apply to the bank for a cut in the loan interest rate, thereby saving financing costs. For example, as the first commercial bank to practice the Equator Principles, Industrial Bank continuously increases support for focus areas such as clean energy, energy conservation, environmental protection, and carbon emission reduction. It has supported 489 emission reduction projects and issued emission reduction loans of CNY 34.16 billion, which strongly support the financing needs of emission reduction projects [15]. Moreover, Huaxin Cement (Huangshi) successfully applied for a preferential loan of RMB 40 million from the Bank of Communications for emission reduction at an interest rate concession of 0.53% and used it for several emission reduction projects [16].

Our literature survey found that although several papers focused on integrated operational and financing decisions, few existing studies incorporate capital constraints, carbon constraints, and financing strategies into the manufacturing/remanufacturing production issues. The capital constraints, carbon credit policy, and emission reduction efficiency of remanufacturing activities have received much more attention. Thus, under the carbon credit policy, we are full of interest about what decisions the capital-constrained manufacturer will make about remanufacturing and carbon emissions with/without a mandatory carbon threshold. Considering a mandatory carbon threshold, how will the capital-constrained manufacturer change optimal remanufacturing and emissions decisions to achieve profit maximization? Given a profit maximization target and a determined carbon threshold, how does the bank determine the optimal preferential interest rate for loans? To address these questions, two profit maximization models are constructed, respectively, from the perspective of the capital-constrained manufacturer and the bank. Specifically, under the carbon credit policy, the bank will offer certain loan interest rate concessions to the capital-constrained manufacturer that meet an agreed carbon threshold. Then, the manufacturer determines the optimal manufacturing/remanufacturing quantities considering the established carbon threshold and interest rate for loans. Finally, based on the methodology of backward induction, the capital-constrained manufacturer’s remanufacturing decision-making process under the carbon credit policy and the bank’s decision-making process of the optimal preferential interest rate for loans are explored through theoretical and numerical analysis.

The remainder of this paper is organized as follows. Section 2 is devoted to a review of the related literature. Materials and research methods are presented in Section 3. The profit maximization models of the capital-constrained manufacturer and the bank are sequentially constructed in Section 4 and Section 5. Finally, conclusions and future research are provided in Section 6.

## 2. Literature Review

The increasingly severe emission reduction situation has prompted more and more manufacturers to incorporate carbon factors into their operational decisions. Correspondingly, from a theoretical perspective, many scholars are devoted to the study of remanufacturing decision issues under different carbon emission policies, such as mandatory carbon caps [17,18,19], carbon tax [20,21,22], and emissions trading policy [23,24,25]. However, these papers consider the carbon factor to be a completely exogenous constraint, ignoring its effect on other factors, such as the loan interest rate mentioned below. Meanwhile, the above literature assumes that the manufacturer’s productive capital is always sufficient, which is a counterfactual condition. In practice, the capital constraint poses difficulties in achieving optimal operational decisions and total profits for manufacturers [1]. Moreover, the capital constraint is also a serious disincentive for enterprises to invest in emissions control and green technologies [26,27]. Thus, some papers explore how capital constraint would affect operational decisions, such as Wang et al. [28], Jin et al. [29], Zhang et al. [30], and Xie et al. [31]. Among them, only the study of [28] involved both emissions constraint and remanufacturing decision, and the results show that the capital constraint severely restricted remanufacturing activities, and the emissions constraint further exacerbated the manufacturer’s capital shortage. 

As capital availability is essential for the feasibility of original optimal operational decisions, manufacturers would seek to address capital constraint issues through external financing. Many scholars have conducted related research, and there is even some literature that involves remanufacturing decisions. For instance, considering bank loans, Wang and Zhang explored a capital-constrained remanufacturing enterprise’s recycling mode selection issue [32]. Wang and Chen successively examined manufacturing/remanufacturing decisions under conditions with capital constraints and bank loans [33]. Introducing deferred payment to a third-party recycler, Li et al. studied a joint decision on recycling, remanufacturing, and reducing emissions under the cap-and-trade and government’s subsidy policies [34]. Moreover, focusing on diverse financing strategies, Zhang et al. investigated the optimal selection of the third-party remanufacturing modes (outsourcing vs. authorization) under the financing portfolio strategy of trade credit and bank loans [35]. Zhang and Chen even compared and analyzed the effects of three financing modes on operational decisions in a capital-constrained remanufacturing supply chain, such as partial trade credit with bank loans (PTC-with-BL), full trade credit with bank loans (FTC-with-BL) and pure bank loans (PBL) [5]. It can be found that the literature mentioned above involving the capital constraint considers merely some traditional financing modes. Meanwhile, this leads to the result that financing strategies only benefit the enterprises’ economic profits and play a minimal role in emissions control. Therefore, based on the consideration of production cost and carbon emission advantages of remanufacturing activities, it is worthwhile to explore the effects of financial innovation on the economic and environmental benefits of the capital-constrained manufacturer.

Many studies show that green finance policy significantly promotes the low-carbon activities of high-pollution and high-emission enterprises [12,36,37]. In addition, taking China as an example, Pan and Dong explored the green finance policy coupling effect on the low-carbon economy by using a dynamic recursive computable general equilibrium model [38]. Lee et al. found that green credit policy could reduce the carbon emission intensity primarily by reducing the coal consumption intensity, and it could enhance carbon emission performance through capital renewal rather than technological innovation [39]. Chen et al. indicated that green credit policies would promote enterprises’ low-carbon technology innovation by increasing their research and development investment and management efficiency [40]. Recently, some scholars have also begun to study the impacts of different green finance policies from the micro-operational dimension as emission reduction targets continue to be penetrated. For instance, Qin et al. found that the green credit policy mainly affects the manufacturer’s total profit through interest rate adjustment, thereby promoting low-carbon technologies in the supply chain [41]. Because the capital-constrained manufacturer can obtain bank loans by pledging carbon assets, Cao and Yu studied a supply chain’s operations and emission reduction issues [42]. Concentrating on operations and green-level decisions of a green supply chain, Fang and Xu conducted a comparative analysis of the performances of pure green credit and partial green credit with pre-payments [43]. Similarly, An et al. discussed the differential effects of the green credit and traditional trade credit on the operational decisions of a supply chain with emission limits [44]. Being involved in remanufacturing decision issues, Fu et al. mainly compared and analyzed the effects of the pure carbon asset pledge financing (PCAPF) strategy and the hybrid carbon asset pledge financing (HCAPF) strategy [6]. Additionally, focusing on the carbon asset attribute, Wang and Wang explored optimal manufacturing and remanufacturing decisions under the carbon credit buy-back policy [1]. Then, based on the study of Wang and Wang [1], Wang et al. examined the effects of different carbon allowance allocation rules of the grandfathering and benchmarking on the performances of the carbon credit buy-back policy [45]. Apparently, there is less literature considering the remanufacturing decision when involving a green credit policy. Moreover, the above studies on green credit policy only consider interest rate concessions and do not consider the corresponding restrictions imposed on enterprises, which is inconsistent with the actual situation.

In summary, emissions constraints, capital constraints, and financing strategies have been integrated into the research of micro-operational decisions, and even some of the literature has begun to focus on the effect of green/carbon finance policies. However, existing studies addressing green credit policy do not consider corresponding restrictions imposed on manufacturers, such as a mandatory carbon threshold. Based on this, even less literature further explores the impact of green credit/carbon credit policy on remanufacturing decisions. Therefore, this paper integrates carbon credit policy into the remanufacturing operation process and studies the capital-constrained manufacturer’s production and emission decision-making issues. Furthermore, we also examined the decision-making process of the bank’s optimal preferential interest rate for loans based on the profit maximization objective. Finally, this paper can enrich remanufacturing decision theory and provide managerial insights and policy implications for capital-constrained manufacturers and banks.

## 3. Materials and Methods

### 3.1. Problem Description and Notations

This study considers a capital-constrained monopolistic manufacturer engaged in the production and sales of both new and remanufactured products in a single period. New and remanufactured products are sold to the same market, and consumers are less willing to pay for remanufactured products than for new ones. Moreover, considering savings advantages in production cost and carbon emissions, unit remanufactured products’ production costs and carbon emissions are lower. Furthermore, the manufacturer is short of capital in manufacturing and remanufacturing activities, and the initial self-owned capital is assumed to be zero to reveal the central issues better. Finally, under the carbon credit policy, a specific carbon threshold *Ê* is set by the bank according to the manufacturer’s historical carbon emissions or carbon quotas in the current year. Then, a loan with a preferential interest rate will be provided to the manufacturer on the premise that total carbon emissions do not exceed the carbon threshold. For the specific level of interest rate concessions, the bank needs to make decisions based on the profit maximization target. Thus, to obtain a bank loan with a lower interest rate, the capital-constrained manufacturer would carry out manufacturing/remanufacturing activities under a mandatory carbon emission constraint, and repay the principal and interest to the bank after the product demands are met. To gain clear insight into our study issues, the models formulated in this paper focus mainly on the sales revenue of new and remanufactured products, manufacturing and remanufacturing costs, the loan interest cost, or revenue. For lucidity and simplicity, decision variables and parameters involved in the models are shown in Table 1.

### 3.2. Assumptions

To better understand our models, the main assumptions adopted by this paper are listed in the following:

**Assumption** **1.** *Consumers’ willingness to pay for new and remanufactured products is heterogeneous and uniformly distributed in [0,δ]. Referring to Ding et al. [46], Dong et al. [47], and Wang et al. [48], the inverse demand functions of new and remanufactured products can be obtained as follows:p_n_* = 1 − *q_n_* − *δq_r_* *and* *p_r_* = *δ*(1 − *q_n_* − *q_r_*).

**Assumption** **2.** *The new and the remanufactured products are produced in separate lines. Due to fewer raw materials and processes, the per unit remanufactured products’ production cost and carbon emissions should be lower, i.e.,c_n_ >c_r_ ande_n_ >e_r_ [1,6,20]. Additionally, we assume that the percentage of carbon emissions per unit of the remanufactured product to the unit of the new product isβ (0 <β* < 1); *thus, for carbon emissions per unit of remanufactured product e_r_* = *βe_n_. Therefore,* *β represents the carbon savings level of remanufactured products; the lower the* *β, the higher the carbon savings level.*

**Assumption** **3.** *Following Wang et al. [48] and Fu et al. [6], the added values of new and remanufactured products are defined as* Δ*_n_* = 1 − *c_n_*(1 + *r*_0_ − *r_p_*), Δ*_r_* = *δ* − *c_r_*(1 + *r*_0_ − *r_p_*), and Δ*_n_* > Δ*_r_* > *δ*Δ*_n_*.

**Assumption** **4.** *The bank is the leader and is committed to maximizing its profit by deciding upon the optimal preferential interest rate for loans under a given carbon threshold. The manufacturer is the follower committed to maximizing total profit by deciding upon the optimal manufacturing/remanufacturing quantities. It is assumed that two classes of decision-makers are risk-neutral, and there is no information asymmetry, i.e., both the bank and the manufacturer share information. Similar assumptions can be found in Wang et al. [49] and Wang and Zhang [50].*

### 3.3. Research Methods

As mentioned above, the bank is the leader, and the manufacturer is the follower. Thus, the game model will be built between the bank and the manufacturer and is solved by using the backward induction method. Additionally, the capital-constrained manufacturer’s profit maximization model is a nonlinear programming problem, and the Kuhn–Tucker Theorem (KKT) will be utilized to obtain its optimality conditions.

## 4. Model Formulation and Analysis

This section mainly explores the impact of the carbon credit policy, that is, under the determined preferential interest rate for loans and carbon threshold, how the capital-constrained manufacturer determines the optimal manufacturing/remanufacturing and carbon emission decisions, as well as the corresponding changes in total profit and consumer surplus. We assume that the carbon threshold (*Ê*) meets *e_n_*(*q_n_ + βq_r_*) ≤ *Ê*. Then, the capital-constrained manufacturer’s profit maximization model function under the carbon credit policy is as follows:

(1) $$\begin{array}{l} \pi_{m}=(1-q_{n}-\delta q_{r})q_{n}+\delta (1-q_{n}-q_{r})q_{r}-(c_{n}q_{n}+c_{r}q_{r})(1+r_{0}-r_{p})\\ s.t.\;\;\;\;\;e_{n}(q_{n}+\beta q_{r})\leq \hat{E} \end{array}$$

Then, the Kuhn–Tucker Theorem (KKT) is utilized to obtain two different solutions: *e_n_*(*q_n_* + *βq_r_*) < *Ê* (Model N) and *e_n_*(*q_n_* + *βq_r_*) *= Ê* (Model Y), respectively.

### 4.1. Model N Availability of Preferential Interest Rate under No Carbon Threshold

The capital-constrained manufacturer’s manufacturing and remanufacturing decisions are investigated under a no carbon threshold in Model N. Thus, the optimal operational strategies are provided in Lemma 1. All proofs are shown in Appendix A.

**Lemma** **1.** *Under no carbon threshold, the optimal manufacturing/remanufacturing quantities and sales prices of new and remanufactured products, total profit, and carbon emissions are given as follows:*$q_{n}^{N*}=\frac{\Delta_{n}-\Delta_{r}}{2(1-\delta )}$, $q_{r}^{N^{*}}=\frac{\Delta_{r}-\delta \Delta_{n}}{2\delta (1-\delta )}$, $p_{n}^{N^{*}}=\frac{2-\Delta_{n}}{2}$, $p_{r}^{N^{*}}=\frac{2\delta -\Delta_{r}}{2}$, $\pi_{m}^{N^{*}}=\frac{\delta \Delta_{n}^{2}-2\delta \Delta_{n}\Delta_{r}+\Delta_{r}^{2}}{4\delta (1-\delta )}$*, and*$E_{m}^{N}=\frac{e_{n}[\delta (\Delta_{n2}-\Delta_{r2})+\beta (\Delta_{r2}-\delta \Delta_{n2})]}{2\delta (1-\delta )}$.

According to Lemma 1, it can be seen that when the carbon threshold cannot play a restraining role and the manufacturer is given a certain interest rate preference, there is no situation where the output of any product type is 0. Moreover, a higher preferential interest rate for loans will only encourage the manufacturer to produce more new products while reducing the remanufacturing quantity. Furthermore, a higher preferential interest rate will always lead to more total carbon emissions while it will bring higher total profit to the manufacturer. To sum up, when there is no mandatory emissions constraint, the preferential interest rate benefits both manufacturers and consumers, but it cannot better promote remanufacturing activities and reduce total carbon emissions.

### 4.2. Model Y Availability of Preferential Interest Rate under a Mandatory Carbon Threshold

In Model Y, when the bank provides a certain interest rate preference for loans, the total carbon emissions of the capital-constrained manufacturer must not exceed the carbon threshold *Ê*. Thus, the manufacturer’s optimal operational strategies are provided in Lemma 2.

**Lemma** **2.** *Under the scenario of a mandatory emissions threshold, the optimal production quantities and sales prices of new and remanufactured products, total profit, and carbon emissions are given as follows: (i) if*$\Delta_{r}>\beta \Delta_{n}$*and*$\hat{E}<{\hat{E}}_{2}$, $q_{n}^{Y*}=0$, $q_{r}^{Y*}=\frac{\hat{E}}{\beta e_{n}}$, $p_{r}^{Y*}=\delta -\frac{\delta \hat{E}}{\beta e_{n}}$, $\pi_{m}^{Y*}=\frac{\beta e_{n}\Delta_{r}\cdot \hat{E}-\delta {\hat{E}}^{2}}{\beta^{2}e_{n}^{2}}$*, and*$E_{m}^{Y}=\hat{E}$*; (ii) if*$\Delta_{r}<\beta \Delta_{n}$*and*$\hat{E}<{\hat{E}}_{4}$, $q_{n}^{Y*}=\frac{\hat{E}}{e_{n}}$, $q_{r}^{Y*}=0$, $p_{n}^{Y*}=1-\frac{\hat{E}}{e_{n}}$, $\pi_{m}^{Y*}=\frac{e_{n}\Delta_{n}\cdot \hat{E}-{\hat{E}}^{2}}{e_{n}^{2}}$*, and*$E_{m}^{Y}=\hat{E}$*; (iii) if*$\Delta_{r}>\beta \Delta_{n}$*and*${\hat{E}}_{2}<\hat{E}<{\hat{E}}_{5}$*, or*$\Delta_{r}<\beta \Delta_{n}$*and*${\hat{E}}_{4}<\hat{E}<{\hat{E}}_{5}$, $q_{n}^{Y*}=\frac{2\delta (1-\beta )\hat{E}-\beta e_{n}(\Delta_{r}-\beta \Delta_{n})}{2e_{n}(\beta^{2}-2\delta \beta +\delta )}$, $q_{r}^{Y*}=\frac{2(\beta -\delta )\hat{E}+e_{n}(\Delta_{r}-\beta \Delta_{n})}{2e_{n}(\beta^{2}-2\delta \beta +\delta )}$, $p_{n}^{Y*}=1-\frac{2\delta (1-\delta )\hat{E}+e_{n}(\delta -\beta )(\Delta_{r}-\beta \Delta_{n})}{2e_{n}(\beta^{2}-2\delta \beta +\delta )}$, $p_{r}^{Y*}=\delta [1-\frac{2\beta (1-\delta )\hat{E}+e_{n}(1-\beta )(\Delta_{r}-\beta \Delta_{n})}{2e_{n}(\beta^{2}-2\delta \beta +\delta )}]$, $\pi_{m}^{Y*}=\frac{4e_{n}[\delta (\Delta_{n}-\Delta_{r})+\beta (\Delta_{r}-\delta \Delta_{n})]\hat{E}-4\delta (1-\delta ){\hat{E}}^{2}+e_{n}^{2}{\left(\Delta_{r}-\beta \Delta_{n}\right)}^{2}}{4e_{n}^{2}(\beta^{2}-2\delta \beta +\delta )}$*, and*$E_{m}^{Y}=\hat{E}$*. Therefore,*${\hat{E}}_{2}=\frac{\beta e_{n}(\Delta_{r}-\beta \Delta_{n})}{2\delta (1-\beta )}$, ${\hat{E}}_{4}=\frac{e_{n}(\beta \Delta_{n}-\Delta_{r})}{2(\beta -\delta )}$*, and*${\hat{E}}_{5}=\frac{e_{n}[\delta (\Delta_{n}-\Delta_{r})+\beta (\Delta_{r}-\delta \Delta_{n})]}{2\delta (1-\delta )}$.

**Figure 1:**
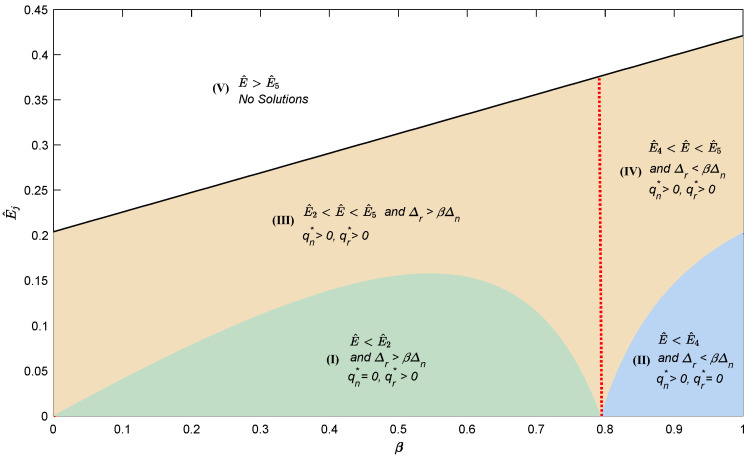
The decision regions of the manufacturer under the carbon credit policy.

To further explore how the decision regions change in Lemma 2 under the carbon credit policy, a numerical example will be conducted. Firstly, to reflect the cost savings of active remanufacturing, the per unit new product production cost is set higher (*c_n_ =* 0.3), and that of the per unit remanufactured product is lower (*c_r_ =* 0.1). Moreover, the general interest rate for loans is set as *r*_0_ = 0.05, and the preferential interest rate for loans needs to satisfy 0 *< r_p_ < r*_0_; so, we let *r_p_ =* 0.01. Then, combining data obtained from investigating some manufacturers and actual practice, the other parameters involved in the model are set as follows: *e_n_ =* 1 and *δ =* 0.65, and the detailed result is shown in Figure 1. It can be found that the optimal operational strategies of the capital-constrained manufacturer are closely related to the carbon threshold, the ratio of the added value of remanufactured products to new products, or the carbon savings level of remanufactured products. It should be noted that the ratio of the added value of two product types or the carbon savings level of remanufactured products is closely related to the loan interest rate preference offered by the bank. To be more specific, when the ratio of the added value of remanufactured products to new products or the carbon savings level of remanufactured products is higher (i.e., 0 < *β < * 0.79), the capital-constrained manufacturer will not produce only new products and not produce only remanufactured products, regardless of changes in the carbon threshold. Conversely, when 0.79 *< β <* 1, the capital-constrained manufacturer will not produce only remanufactured products and not produce only new products, regardless of changes in the carbon threshold. Moreover, when the carbon threshold exceeds a certain value (i.e., *Ê*_2_ or *Ê*_4_), the capital-constrained manufacturer will always produce both new and remanufactured products within a reasonable range (i.e., *Ê < Ê*_5_). However, no matter how the manufacturer adjusts the production strategy, total carbon emissions will always equal the carbon threshold with the profit maximization goal.

**Figure 2:**
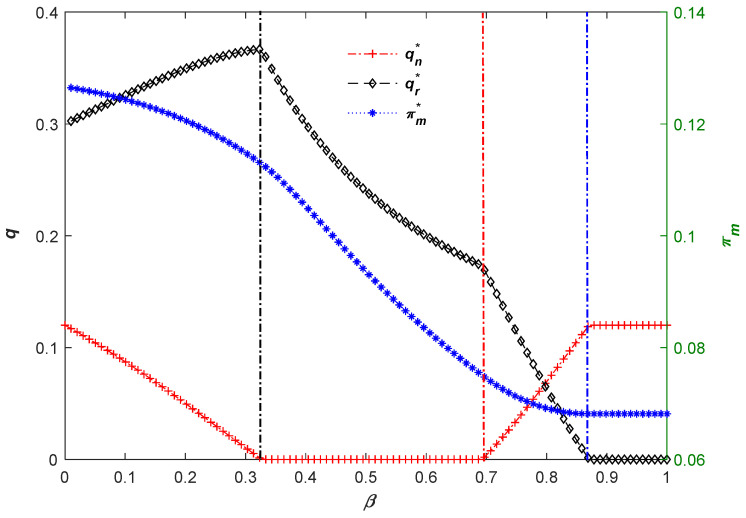
The manufacturer’s decision process and total profit under carbon credit policy.

Next, we set *Ê =* 0.12 and then conduct a numerical analysis to further explore the capital-constrained manufacturer’s operational strategies and total profits in different decision regions. As seen in Figure 2, there are three critical values which are *β =* 0.33, *β =* 0.69, and *β =* 0.87, and decision regions will sequentially change from (III), (I), (III), and (IV) to (Ⅱ) with a change in *β*. Overall, the rising *β* will increase the manufacturing quantity and decrease the remanufacturing quantity. The difference is that when the carbon savings of the remanufactured products are high enough (i.e., *β* < 0.33), the rising *β* will cause the capital-constrained manufacturer to increase the remanufacturing quantity further while reducing t, the manufacturing quantity. In other words, the advantage of carbon savings will always induce the manufacturer to produce remanufactured products under a given carbon threshold. Correspondingly, when the carbon savings level of remanufactured products is lower (i.e., *β* > 0.79), the manufacturing quantity gradually exceeds the remanufacturing quantity. However, no matter how the capital-constrained manufacturer’s production strategy changes, the rising *β* is not always conducive to improving total profit.

Furthermore, the relationship between the capital-constrained manufacturer’s production quantities, sales price, total profit, carbon threshold, and preferential interest rate for loans will be presented in Propositions 1–3. It should be noted that, as mentioned in Lemma 2, when the capital-constrained manufacturer only produces the new or remanufactured product, the optimal operational decision is not associated with the preferential interest rate for loans. Therefore, we only consider the situations where the manufacturer produces new and remanufactured products when analyzing the impact of the preferential interest rate for loans.

**Proposition** **1.** *(i) When the capital-constrained manufacturer produces only one type of product,*$\frac{\partial q_{n}^{Y*}}{\partial \hat{E}}>0$*or*$\frac{\partial q_{r}^{Y*}}{\partial \hat{E}}>0$*; when the capital-constrained manufacturer produces the new and remanufactured products,*$\frac{\partial q_{n}^{Y*}}{\partial \hat{E}}>0$*, and if*β>δ*, then*$\frac{\partial q_{r}^{Y*}}{\partial \hat{E}}>0$*; otherwise,*$\frac{\partial q_{r}^{Y*}}{\partial \hat{E}}<0$*; (ii)*$\frac{\partial p_{n}^{Y*}}{\partial \hat{E}}<0$, $\frac{\partial p_{r}^{Y*}}{\partial \hat{E}}<0$.

Proposition 1 implies that the manufacturer’s manufacturing quantity will always decline as the carbon threshold decreases. The decline in the remanufacturing quantity with the reduction in the carbon threshold occurs when the manufacturer produces merely remanufactured products. In the case of producing both new and remanufactured products, only when the carbon savings level of remanufactured products or consumers’ willingness to pay for remanufactured products is higher (i.e., β<δ), does the manufacturer increase the remanufacturing quantity as the carbon threshold decreases. This also means that the declining carbon threshold can effectively promote remanufacturing activities and appropriately control the total carbon emissions. Consequently, a lower carbon threshold will always force the capital-constrained manufacturer to raise the sales prices of both product types to maximize total profit.

**Proposition** **2.** *The capital-constrained manufacturer produces both new and remanufactured products (i) if*$\beta <\frac{c_{r}}{c_{n}}$, $\frac{\partial q_{n}^{Y*}}{\partial r_{p}}<0$, $\frac{\partial q_{r}^{Y*}}{\partial r_{p}}>0$, $\frac{\partial p_{n}^{Y*}}{\partial r_{p}}<0$*, and*$\frac{\partial p_{r}^{Y*}}{\partial r_{p}}<0$*; (ii) if*$\frac{c_{r}}{c_{n}}<\beta <\delta$, $\frac{\partial q_{n}^{Y*}}{\partial r_{p}}>0$, $\frac{\partial q_{r}^{Y*}}{\partial r_{p}}<0$, $\frac{\partial p_{n}^{Y*}}{\partial r_{p}}>0$*, and*$\frac{\partial p_{r}^{Y*}}{\partial r_{p}}>0$*; (iii) if*β>δ, $\frac{\partial q_{n}^{Y*}}{\partial r_{p}}>0$, $\frac{\partial q_{r}^{Y*}}{\partial r_{p}}<0$, $\frac{\partial p_{n}^{Y*}}{\partial r_{p}}<0$*, and*$\frac{\partial p_{r}^{Y*}}{\partial r_{p}}>0$.

Proposition 2 illustrates that the effects of the preferential interest rate on the capital-constrained manufacturer’s production quantities and sales prices are closely related to the range of the carbon savings level of remanufactured products. The rising preferential interest rate will encourage the manufacturer to further increase the remanufacturing quantity when the carbon savings level of remanufactured products is high enough (i.e., $\beta <\frac{c_{r}}{c_{n}}$). Additionally, the declining financing cost will induce the manufacturer to benefit consumers by lowering the sales prices of new and remanufactured products. When the carbon savings level of the remanufactured product is low (i.e., $\beta >\frac{c_{r}}{c_{n}}$), the rising preferential interest rate will increase the manufacturing quantity and decrease the remanufacturing quantity. Meanwhile, the increasing sales price of remanufactured products is also not conducive to remanufacturing activities.

**Proposition** **3.** *(i)*$\frac{\partial \pi_{m}^{Y*}}{\partial \hat{E}}>0$*; (ii) if*$r_{p}>r_{p}^{m}$*, then*$\frac{\partial \pi_{m}^{Y*}}{\partial r_{p}}>0$*; otherwise,*$\frac{\partial \pi_{m}^{Y*}}{\partial r_{p}}<0$, *in which*$r_{p}^{m}=\frac{e_{n}{\left(c_{r}-\beta c_{n}\right)}^{2}(1+r_{0})+e_{n}(\beta -\delta )(c_{r}-\beta c_{n})-2[\delta (c_{n}-c_{r})-\beta (\delta c_{n}-c_{r})]\hat{E}}{e_{n}{\left(c_{r}-\beta c_{n}\right)}^{2}}$.

Proposition 3 shows that under the carbon credit policy, the carbon threshold is always positively correlated with the capital-constrained manufacturer’s total profit. It also indicates that the higher total profit is often at the expense of heavy carbon emissions. This is mainly because the rising carbon threshold always leads to a higher manufacturing quantity, and thus the high profitability of new products brings a higher total profit. However, the manufacturer’s total profit initially decreases and then increases as the preferential interest rate rises. That is, an increase in the preferential interest rate is not necessarily conducive to an increase in the capital-constrained manufacturer’s total profit. In addition, to control the total carbon emissions through a lower carbon threshold, the bank needs to improve the preferential interest rate for loans to guarantee the manufacturer’s total profit within a higher range of *r_p_*.

### 4.3. Effects on Consumer Surplus

This subsection mainly explores the effects of the carbon credit policy on consumer surplus. Referring to Ding et al. [46] and Wang et al. [48], the consumer surplus function is shown as follows:

(2) $$\pi_{c}=\frac{q_{n}^{2}+\delta q_{r}^{2}+2\delta q_{n}q_{r}}{2}=\frac{4\delta (1-\delta ){\hat{E}}^{2}+e_{n}^{2}{\left(\Delta_{r}-\beta \Delta_{n}\right)}^{2}}{8e_{n}^{2}(\beta^{2}-2\delta \beta +\delta )}$$

**Proposition** **4.** *(i)*$\frac{\partial \pi_{c}}{\partial \hat{E}}>0$; *(ii) if*$r_{p}>r_{p}^{c}$, *then*$\frac{\partial \pi_{c}}{\partial r_{p}}>0$*; otherwise,*$\frac{\partial \pi_{c}}{\partial r_{p}}<0$*, in which*$r_{p}^{c}=\frac{\left(c_{r}-\beta c_{n})(1+r_{0})+(\beta -\delta \right)}{c_{r}-\beta c_{n}}$.

Proposition 4 indicates that the consumer surplus always has a positive correlation with the carbon threshold under the carbon credit policy, which is also consistent with Proposition 1. A higher carbon threshold will always lead to a lower sales price. Additionally, the consumer surplus initially decreases and then increases as the preferential interest rate rises. The difference is that the carbon threshold does not affect the critical value. Furthermore, only when $r_{p}>r_{p}^{c}$, does the rising preferential interest rate benefit both capital-constrained manufacturers and consumers since $r_{p}^{c}>r_{p}^{m}$. Correspondingly, only when $r_{p}<r_{p}^{m}$ does the declining preferential interest rate benefit both capital-constrained manufacturers and consumers.

## 5. The Bank’s Optimal Decision

As a leader, the bank designs and adjusts the carbon credit policy according to the manufacturer’s decision feedback. This section mainly explores how the bank decides upon the optimal preferential interest rate for loans under a given carbon threshold to maximize its total profit. In addition, this section only considers the situation where the carbon threshold plays a mandatory restraining role. It can be seen from Lemma 2 that when the capital-constrained manufacturer only produces one type of product, the production quantity has no correlation with the preferential interest rate for loans. Meanwhile, the bank’s total profit is always inversely correlated with the preferential interest rate and positively correlated with the carbon threshold. Therefore, this section only considers the situation where the capital-constrained manufacturer produces both new and remanufactured products. Then, the bank’s total profit maximization model function can be written as follows:

(3) $$\begin{array}{l} \pi_{b}=(c_{n}q_{n}^{Y*}+c_{r}q_{r}^{Y*})(r_{0}-r_{p})\\ =\frac{2\hat{E}[\delta (c_{n}-c_{r})-\beta (\delta c_{n}-c_{r})]+e_{n}(c_{r}-\beta c_{n})(\Delta_{r}-\beta \Delta_{n})}{2e_{n}(\beta^{2}-2\delta \beta +\delta )}(r_{0}-r_{p}) \end{array}$$

**Lemma** **3.** *Under a given carbon threshold, the bank’s optimal preferential interest rate for loans is*$r_{p}^{*}=\frac{e_{n}{\left(c_{r}-\beta c_{n}\right)}^{2}(1+2r_{0})+e_{n}(c_{r}-\beta c_{n})(\beta -\delta )-2\hat{E}[\delta (c_{n}-c_{r})-\beta (\delta c_{n}-c_{r})]}{2e_{n}{\left(c_{r}-\beta c_{n}\right)}^{2}}$, *in which*$\frac{e_{n}{\left(c_{r}-\beta c_{n}\right)}^{2}+e_{n}(c_{r}-\beta c_{n})(\beta -\delta )}{2[\delta (c_{n}-c_{r})-\beta (\delta c_{n}-c_{r})]}<\hat{E}<\frac{e_{n}{\left(c_{r}-\beta c_{n}\right)}^{2}(1+2r_{0})+e_{n}(c_{r}-\beta c_{n})(\beta -\delta )}{2[\delta (c_{n}-c_{r})-\beta (\delta c_{n}-c_{r})]}$.

It can be seen from Lemma 3 that for the sake of profit maximization, the bank will determine the optimal preferential interest rate based on factors such as carbon threshold, etc. Moreover, only when the carbon threshold is within a certain range can the bank provide a preferential interest rate to a capital-constrained manufacturer. Then, according to Lemma 3, we can easily obtain Propositions 5–7.

**Proposition** **5.** *When*$0<\beta <\frac{2\hat{E}(c_{n}-c_{r})+e_{n}c_{r}}{(2\hat{E}+e_{n})c_{n}}$, $\frac{\partial r_{p}^{*}}{\partial \delta }<0$; *when*$\frac{2\hat{E}(c_{n}-c_{r})+e_{n}c_{r}}{(2\hat{E}+e_{n})c_{n}}<\beta <1$, $\frac{\partial r_{p}^{*}}{\partial \delta }>0$.

It can be obtained from Proposition 5 that when the carbon threshold remains unchanged under the carbon credit policy, consumers’ increasingly stronger willingness to pay for remanufactured products can better guide the bank to provide a higher preferential interest rate if the carbon savings level of remanufacturing activities is low. This implies that in an environment where consumers’ environmental awareness is constantly increasing, the carbon credit policy is more beneficial to the manufacturer whose carbon savings level for remanufactured products cannot be effectively enhanced and is more conducive to the expansion of remanufacturing categories or quantities. Additionally, when the carbon savings level of remanufactured products is larger, consumers’ increasingly stronger willingness to pay for remanufactured products will lead to a lower preferential interest rate from the bank. Then, the bank can also better provide low-cost loans to the manufacturer with low acceptance of remanufactured products through the carbon credit policy, so as to further promote remanufacturing activities.

**Proposition** **6.** *When*$\frac{c_{r}}{c_{n}}<\beta <\frac{4\hat{E}\cdot \delta c_{n}(c_{n}-c_{r})-2\hat{E}\cdot c_{r}(\delta c_{n}-c_{r})+e_{n}c_{r}(\delta c_{n}-c_{r})}{\left(2\hat{E}+e_{n})c_{n}(\delta c_{n}-c_{r}\right)}$, $\frac{\partial r_{p}^{*}}{\partial \beta }>0$; *when*$0<\beta <\frac{c_{r}}{c_{n}}$*or*$\frac{4\hat{E}\cdot \delta c_{n}(c_{n}-c_{r})-2\hat{E}\cdot c_{r}(\delta c_{n}-c_{r})+e_{n}c_{r}(\delta c_{n}-c_{r})}{\left(2\hat{E}+e_{n})c_{n}(\delta c_{n}-c_{r}\right)}<\beta <1$, $\frac{\partial r_{p}^{*}}{\partial \beta }<0$.

From Proposition 6, it can be found that when the carbon threshold remains unchanged under the carbon credit policy, the relationship between the preferential interest rate and the carbon savings level of remanufactured products depends on the value of *β*. Relatively speaking, when the carbon savings level of remanufactured products is too low or too high, further improving the carbon savings level (i.e., a decrease in *β*) can better guide the bank to provide a higher preferential interest rate, thereby providing the capital-constrained manufacturer with better financial services. Ultimately, on the premise that the total carbon emissions remain unchanged, a higher preferential interest rate encourages more or a larger range of remanufacturing activities by manufacturers while the bank achieves profit maximization.

**Proposition** **7.** *(i)*$\frac{\partial r_{p}^{*}}{\partial \hat{E}}<0$; *(ii)*$\frac{\partial \pi_{b}^{*}}{\partial \hat{E}}>0$.

Proposition 7 demonstrates that the bank’s optimal preferential interest rate is inversely correlated with the carbon threshold, while the bank’s total profit is positively correlated with the carbon threshold. This also means that the rising preferential interest rate for loans is not always conducive to the bank’s total profit. Additionally, by combining Propositions 3 and 4, it can be found that although the rising carbon threshold is not conducive to controlling total carbon emissions, it always benefits the capital-constrained manufacturer, consumers, and banks. Therefore, if a stricter carbon threshold is needed to control the total carbon emissions, the government should provide appropriate subsidies to the above three participants or one of them to compensate for the possible loss in profits. Meanwhile, to further promote the capital-constrained manufacturer’s remanufacturing activities, it is necessary to enhance the carbon savings level of remanufactured products or the consumers’ willingness to pay for remanufactured products, as stated in Proposition 1.

## 6. Conclusions

In this work, we mainly studied a capital-constrained manufacturer’s remanufacturing and emissions decisions in a single period under the carbon credit policy. Firstly, a profit maximization model was formulated, and the effects of the carbon credit policy on the manufacturer’s manufacturing/remanufacturing decisions, total carbon emissions, and total profit were investigated. Then, we constructed the profit maximization model of the bank to explore the optimal preferential interest rate for loans based on the manufacturer’s decision feedback under a given carbon threshold. Our results provide the following conclusions:

(1) Under the carbon credit policy, an excessively high carbon threshold will not be conducive to promoting the capital-constrained manufacturer to carry out remanufacturing activities and controlling total carbon emissions. When the carbon threshold can play a mandatory restraining role, the capital-constrained manufacturer’s optimal operational strategies are closely related to the carbon threshold and the preferential interest rate for loans, etc. Furthermore, no matter how the manufacturer adjusts its production strategies, total carbon emissions will always be equal to the carbon threshold to achieve profit maximization.
(2) Under the carbon credit policy, when the carbon savings level of remanufactured products is high enough, the declining carbon threshold or the rising preferential interest rate for loans will increase the production quantity of remanufactured products and decrease the production quantity of new products. This also means that, at this time, the carbon credit policy can achieve both an increase in remanufacturing quantity and a reduction in total carbon emissions. Meanwhile, the declining financing cost will induce the manufacturer to benefit consumers by lowering the sales prices of new and remanufactured products.
(3) Under the carbon credit policy, the optimal preferential interest rate for loans is inversely correlated with the carbon threshold. When the carbon threshold remains unchanged, the increase in consumers’ willingness to pay for remanufactured products can better guide the bank to provide a higher preferential interest rate if the carbon savings level of remanufactured products is low. Furthermore, if the carbon savings level of remanufactured products is too high, the further improvement in the carbon savings level (i.e., a decrease in *β*) can better guide the bank to provide a higher preferential interest rate, thereby providing better financial services to promote manufacturers’ remanufacturing activities. Ultimately, on the premise that the total carbon emissions remain unchanged, a higher preferential interest rate encourages more or a larger range of remanufacturing activities by manufacturers while the bank achieves profit maximization.

According to the above conclusions, we can obtain the following managerial insights and policy implications. From the manufacturer’s perspective, a higher carbon savings level of remanufactured products can better bring a positive effect of the carbon credit policy on remanufacturing and emissions decisions. Then, it is very beneficial for them to address enhancing the carbon saving level of remanufactured products, such as improving recycled product quality and remanufacturing technology innovation. From the banks’ perspective, firstly, the carbon threshold should not be too high, otherwise the carbon credit policy will not achieve the expected effect. Moreover, in an environment where consumers’ environmental awareness is constantly increasing, a more favorable carbon credit policy should be adopted to be beneficial for the expansion of remanufacturing categories or quantities. Finally, to better ensure that the carbon credit policy is economically and environmentally balanced, some appropriate government subsidies should be provided to manufacturers and/or banks.

There are a few limitations in this study. First, the stochastic product demand should be considered, and we should also address the situation where the capital-constrained manufacturer cannot repay the loan principal and interest at the end of the period and faces bankruptcy. Additionally, more financing modes could be covered, and the comparative analysis of the effects on the low-carbon operations and total carbon emissions could be conducted.

## Figures and Tables

**Table 1:** Notations for decision variables and parameters.

**Decision Variables**	**Descriptions**
*q_n_*, *q_r_*	Manufacturing/Remanufacturing quantities
*r_p_*	Preferential interest rate for loans
**Relevant parameters**	**Descriptions**
*p_n_*, *p_r_*	Sales price per unit of new and remanufactured products, *p_n_* > *p_r_*
*c_n_*, *c_r_*	Production cost per unit of new and remanufactured products, *c_n_ > c_r_*
*δ*	Consumers’ willingness to pay for remanufactured products, 0 < *δ* < 1
*e_n_*	Carbon emissions of unit of new product
*β*	The percentage of carbon emissions per unit of remanufactured product to unit of new product, 0 < *β* < 1
*Ê*	Carbon threshold
*r*_0_	General interest rate for loans
*π_m_*	Manufacturer’s total profit
*E_m_*	Manufacturer’s total carbon emissions
*π_c_*	Consumer surplus
*π_b_*	Bank’s total profit

## Data Availability

The dataset used and/or analyzed in this study is available from the corresponding author upon reasonable request.

## Appendix A

**Proof of Lemma** **1.** According to Decision Model (1), the corresponding Lagrangian function can be written as follows:

$$\begin{array}{l} L(q_{n},q_{r},\lambda_{1},\lambda_{2},\lambda_{3})=(1-q_{n}-\delta q_{r})q_{n}+\delta (1-q_{n}-q_{r})q_{r}-(c_{n}q_{n}+c_{r}q_{r})(1+r_{0}-r_{p})\\ +\lambda_{1}[\hat{E}-e_{n}(q_{n}+\beta q_{r})]+\lambda_{2}q_{n}+\lambda_{3}q_{r} \end{array}$$

Then, the optimal values of decision variables must satisfy the following equations according to KKT conditions.

$$\frac{\partial \pi_{m}}{\partial q_{n}}=1-c_{n}(1+r_{0}-r_{p})-2q_{n}-2\delta q_{r}-\lambda_{1}e_{n}+\lambda_{2}=\Delta_{n}-2q_{n}-2\delta q_{r}-\lambda_{1}e_{n}+\lambda_{2}=0$$

$$\frac{\partial \pi_{m}}{\partial q_{r}}=\delta -c_{r}(1+r_{0}-r_{p})-2\delta q_{n}-2\delta q_{r}-\lambda_{1}\beta e_{n}+\lambda_{3}=\Delta_{r}-2\delta q_{n}-2\delta q_{r}-\lambda_{1}\beta e_{n}+\lambda_{3}=0$$

$$\lambda_{1}[\hat{E}-e_{n}(q_{n}+\beta q_{r})]=0$$

(1) If $\lambda_{1}=0,\lambda_{2}>0,\lambda_{3}=0$ (i.e., $q_{n}=0,q_{r}>0$), we have $\Delta_{n}-2\delta q_{r}+\lambda_{2}=0$ and $\Delta_{r}-2\delta q_{r}=0$. Then, we can obtain $q_{r}=\frac{\Delta_{r}}{2\delta }$ and $\lambda_{2}=\Delta_{r}-\Delta_{n}<0$. Therefore, there is no solution. (2) If $\lambda_{1}=0,\lambda_{2}=0,\lambda_{3}>0$ (i.e., $q_{n}>0,q_{r}=0$), we have $\Delta_{n}-2q_{n}=0$ and $\Delta_{r}-2\delta q_{n}+\lambda_{3}=0$. Then, we can obtain $q_{r}=\frac{\Delta_{n}}{2}$ and $\lambda_{3}=\delta \Delta_{n}-\Delta_{r}<0$. Therefore, there is no solution. (3) If $\lambda_{1}=0,\lambda_{2}=0,\lambda_{3}=0$ (i.e., $q_{n}>0,q_{r}>0$), we have $\Delta_{n}-2q_{n}-2\delta q_{r}=0$ and $\Delta_{r}-2\delta q_{n}-2\delta q_{r}=0$. Then, we can obtain $q_{n}^{N*}=\frac{\Delta_{n}-\Delta_{r}}{2(1-\delta )}$, $q_{r}^{N*}=\frac{\Delta_{r}-\delta \Delta_{n}}{2\delta (1-\delta )}$, $p_{n}^{N*}=\frac{2-\Delta_{n}}{2}$, $p_{r}^{N*}=\frac{2\delta -\Delta_{r}}{2}$, $\pi_{m}^{N*}=\frac{\delta \Delta_{n}^{2}-2\delta \Delta_{n}\Delta_{r}+\Delta_{r}^{2}}{4\delta (1-\delta )}$, and $E_{m}^{N}=\frac{e_{n}[\delta (\Delta_{n}-\Delta_{r})+\beta (\Delta_{r}-\delta \Delta_{n})]}{2\delta (1-\delta )}$. Thus, Lemma 1 is proven. □

**Proof of Lemma** **2.** According to the above KKT conditions, we carry out the following discussion: (1) If $\lambda_{1}>0,\lambda_{2}>0,\lambda_{3}=0$ (i.e., $q_{n}=0,q_{r}>0$), we have $\Delta_{n}-2\delta q_{r}-\lambda_{1}e_{n}+\lambda_{2}=0$, $\Delta_{r}-2\delta q_{r}-\lambda_{1}\beta e_{n}=0$, and $\beta e_{n}\cdot q_{r}=\hat{E}$. Then, we can obtain $q_{r}=\frac{\hat{E}}{\beta e_{n}}$, $\lambda_{1}=\frac{\beta e_{n}\cdot \Delta_{r}-2\delta \hat{E}}{\beta^{2}e_{n}^{2}}$, and $\lambda_{2}=\frac{\beta e_{n}(\Delta_{r}-\beta \Delta_{n})-2\delta (1-\beta )\hat{E}}{\beta^{2}e_{n}}$. Thus, if $\Delta_{r}<\beta \Delta_{n}$, there always exists $\lambda_{2}<0$; there is no solution. If $\Delta_{r}>\beta \Delta_{n}$, then $\lambda_{1}>0\Rightarrow \hat{E}<\frac{\beta e_{n}\cdot \Delta_{r}}{2\delta }={\hat{E}}_{1}$ and $\lambda_{2}>0\Rightarrow \hat{E}<\frac{\beta e_{n}(\Delta_{r}-\beta \Delta_{n})}{2\delta (1-\beta )}={\hat{E}}_{2}$. Therefore, ${\hat{E}}_{1}-{\hat{E}}_{2}=\frac{\beta^{2}e_{n}(\Delta_{n}-\Delta_{r})}{2\delta (1-\beta )}>0$, i.e., ${\hat{E}}_{1}>{\hat{E}}_{2}$. To sum up, when $\Delta_{r}>\beta \Delta_{n}$ and $\hat{E}<{\hat{E}}_{2}$, we have $q_{n}^{Y*}=0$, $q_{r}^{Y*}=\frac{\hat{E}}{\beta e_{n}}$, $p_{r}^{Y*}=\delta -\frac{\delta \hat{E}}{\beta e_{n}}$, $E_{m}^{Y}=\hat{E}$, and $\pi_{m}^{Y*}=\frac{\beta e_{n}\Delta_{r}\cdot \hat{E}-\delta {\hat{E}}^{2}}{\beta^{2}e_{n}^{2}}$. (2) If $\lambda_{1}>0,\lambda_{2}=0,\lambda_{3}>0$ (i.e., $q_{n}>0,q_{r}=0$), we have $\Delta_{n}-2q_{n}-\lambda_{1}e_{n}=0$, $\Delta_{r}-2\delta q_{n}-\lambda_{1}\beta e_{n}+\lambda_{3}=0$, and $e_{n}q_{n}=\hat{E}$. Then, we can obtain $q_{n}=\frac{\hat{E}}{e_{n}}$, $\lambda_{1}=\frac{e_{n}\cdot \Delta_{n}-2\hat{E}}{e_{n}^{2}}$, and $\lambda_{3}=\frac{2(\delta -\beta )\hat{E}-e_{n}(\Delta_{r}-\beta \Delta_{n})}{e_{n}}$. Thus, if $\Delta_{r}>\delta \Delta_{n}>\beta \Delta_{n}$, then $\lambda_{1}>0\Rightarrow \hat{E}<\frac{e_{n}\Delta_{n}}{2}={\hat{E}}_{3}$ and $\lambda_{3}>0\Rightarrow \hat{E}>\frac{e_{n}(\Delta_{r}-\beta \Delta_{n})}{2(\delta -\beta )}={\hat{E}}_{4}$. Therefore, ${\hat{E}}_{3}-{\hat{E}}_{4}=\frac{e_{n}(\delta \Delta_{n}-\Delta_{r})}{2(\delta -\beta )}<0$, i.e., ${\hat{E}}_{3}<{\hat{E}}_{4}$. The conditions that make $\lambda_{1}>0$ and $\lambda_{3}>0$ hold are mutually contradictory, and thus there is no solution. If $\Delta_{r}>\beta \Delta_{n}>\delta \Delta_{n}$, there always exists $\lambda_{3}<0$; there is no solution. If $\beta \Delta_{n}>\Delta_{r}>\delta \Delta_{n}$, then $\lambda_{1}>0\Rightarrow \hat{E}<\frac{e_{n}\Delta_{n}}{2}={\hat{E}}_{3}$ and $\lambda_{3}>0\Rightarrow \hat{E}<\frac{e_{n}(\beta \Delta_{n}-\Delta_{r})}{2(\beta -\delta )}={\hat{E}}_{4}$. Therefore, ${\hat{E}}_{3}-{\hat{E}}_{4}=\frac{e_{n}(\delta \Delta_{n}-\Delta_{r})}{2(\delta -\beta )}>0$, i.e., ${\hat{E}}_{3}>{\hat{E}}_{4}$. To sum up, when $\Delta_{r}<\beta \Delta_{n}$ and $\hat{E}<{\hat{E}}_{4}$, we have $q_{n}^{Y*}=\frac{\hat{E}}{e_{n}}$, $q_{r}^{Y*}=0$, $p_{n}^{Y*}=1-\frac{\hat{E}}{e_{n}}$, $E_{m}^{Y}=\hat{E}$, and $\pi_{m}^{Y*}=\frac{e_{n}\Delta_{n}\cdot \hat{E}-{\hat{E}}^{2}}{e_{n}^{2}}$. (3) If $\lambda_{1}>0,\lambda_{2}=0,\lambda_{3}=0$ (i.e., $q_{n}>0,q_{r}>0$), we have $\Delta_{n}-2q_{n}-2\delta q_{r}-\lambda_{1}e_{n}=0$, $\Delta_{r}-2\delta q_{n}-2\delta q_{r}-\lambda_{1}\beta e_{n}=0$ and $\lambda_{1}[\hat{E}-e_{n}(q_{n}+\beta q_{r})]=0$. Then, we can obtain $q_{n}=\frac{2\delta (1-\beta )\hat{E}-\beta e_{n}(\Delta_{r}-\beta \Delta_{n})}{2e_{n}(\beta^{2}-2\delta \beta +\delta )}$, $q_{r}=\frac{2(\beta -\delta )\hat{E}+e_{n}(\Delta_{r}-\beta \Delta_{n})}{2e_{n}(\beta^{2}-2\delta \beta +\delta )}$, and $\lambda_{1}=\frac{e_{n}[\delta (\Delta_{n}-\Delta_{r})+\beta (\Delta_{r}-\delta \Delta_{n})]-2\delta (1-\delta )\hat{E}}{e_{n}^{2}(\beta^{2}-2\delta \beta +\delta )}$. Thus, if $\Delta_{r}>\beta \Delta_{n}>\delta \Delta_{n}$, there always exists $q_{r}>0$. Additionally, we have $q_{n}>0\Rightarrow \hat{E}>\frac{\beta e_{n}(\Delta_{r}-\beta \Delta_{n})}{2\delta (1-\beta )}={\hat{E}}_{2}$ and $\lambda_{1}>0\Rightarrow \hat{E}<\frac{e_{n}[\delta (\Delta_{n}-\Delta_{r})+\beta (\Delta_{r}-\delta \Delta_{n})]}{2\delta (1-\delta )}={\hat{E}}_{5}$. Therefore, ${\hat{E}}_{2}-{\hat{E}}_{5}=\frac{e_{n}(\delta -2\delta \beta +\beta^{2})(\Delta_{r}-\Delta_{n})}{2\delta (1-\delta )(1-\beta )}<0$, i.e., ${\hat{E}}_{2}<{\hat{E}}_{5}$. So, when $\Delta_{r}>\beta \Delta_{n}>\delta \Delta_{n}$ and ${\hat{E}}_{2}<\hat{E}<{\hat{E}}_{5}$, the problem has the above optimal solutions. If $\Delta_{r}>\delta \Delta_{n}>\beta \Delta_{n}$, we have $q_{n}>0\Rightarrow \hat{E}>\frac{\beta e_{n}(\Delta_{r}-\beta \Delta_{n})}{2\delta (1-\beta )}={\hat{E}}_{2}$, $q_{r}>0\Rightarrow \hat{E}<\frac{e_{n}(\Delta_{r}-\beta \Delta_{n})}{2(\delta -\beta )}={\hat{E}}_{4}$, and $\lambda_{1}>0\Rightarrow \hat{E}<\frac{e_{n}[\delta (\Delta_{n}-\Delta_{r})+\beta (\Delta_{r}-\delta \Delta_{n})]}{2\delta (1-\delta )}={\hat{E}}_{5}$. Therefore, ${\hat{E}}_{2}-{\hat{E}}_{4}=\frac{\left(2\delta \beta -\delta -\beta^{2})e_{n}(\Delta_{r}-\beta \Delta_{n}\right)}{2\delta (1-\beta )(\delta -\beta )}<0$ and ${\hat{E}}_{4}-{\hat{E}}_{5}=\frac{\left(\delta +\beta^{2}-2\delta \beta )e_{n}(\Delta_{r}-\delta \Delta_{n}\right)}{2\delta (1-\delta )(\delta -\beta )}>0$, i.e., ${\hat{E}}_{2}<{\hat{E}}_{5}<{\hat{E}}_{4}$. So, when $\Delta_{r}>\delta \Delta_{n}>\beta \Delta_{n}$ and ${\hat{E}}_{2}<\hat{E}<{\hat{E}}_{5}$, the problem has the above optimal solutions. If $\beta \Delta_{n}>\Delta_{r}>\delta \Delta_{n}$, there always exists $q_{n}>0$. In addition, we have $q_{r}>0\Rightarrow \hat{E}>\frac{e_{n}(\beta \Delta_{n}-\Delta_{r})}{2(\beta -\delta )}={\hat{E}}_{4}$. Therefore, ${\hat{E}}_{4}-{\hat{E}}_{5}=\frac{\left(\delta +\beta^{2}-2\delta \beta )e_{n}(\Delta_{r}-\delta \Delta_{n}\right)}{2\delta (1-\delta )(\delta -\beta )}<0$, i.e., ${\hat{E}}_{4}<{\hat{E}}_{5}$. So, when $\Delta_{r}<\beta \Delta_{n}$ and ${\hat{E}}_{4}<\hat{E}<{\hat{E}}_{5}$, the problem has the above optimal solutions. To sum up, when $\Delta_{r}>\beta \Delta_{n}$ and ${\hat{E}}_{2}<\hat{E}<{\hat{E}}_{5}$, or $\Delta_{r}<\beta \Delta_{n}$ and ${\hat{E}}_{4}<\hat{E}<{\hat{E}}_{5}$, we have $q_{n}^{Y*}=\frac{2\delta (1-\beta )\hat{E}-\beta e_{n}(\Delta_{r}-\beta \Delta_{n})}{2e_{n}(\beta^{2}-2\delta \beta +\delta )}$, $q_{r}^{Y*}=\frac{2(\beta -\delta )\hat{E}+e_{n}(\Delta_{r}-\beta \Delta_{n})}{2e_{n}(\beta^{2}-2\delta \beta +\delta )}$, $p_{n}^{Y*}=1-\frac{2\delta (1-\delta )\hat{E}+e_{n}(\delta -\beta )(\Delta_{r}-\beta \Delta_{n})}{2e_{n}(\beta^{2}-2\delta \beta +\delta )}$, $p_{r}^{Y*}=\delta [1-\frac{2\beta (1-\delta )\hat{E}+e_{n}(1-\beta )(\Delta_{r}-\beta \Delta_{n})}{2e_{n}(\beta^{2}-2\delta \beta +\delta )}]$, $\pi_{m}^{Y*}=\frac{4e_{n}[\delta (\Delta_{n}-\Delta_{r})+\beta (\Delta_{r}-\delta \Delta_{n})]\hat{E}-4\delta (1-\delta ){\hat{E}}^{2}+e_{n}^{2}{\left(\Delta_{r}-\beta \Delta_{n}\right)}^{2}}{4e_{n}^{2}(\beta^{2}-2\delta \beta +\delta )}$, and $E_{m}^{Y}=\hat{E}$. Therefore, Lemma 2 is proven. □

**Proof of Proposition** **1.** According to the expressions of $q_{n}^{Y*}$, $q_{r}^{Y*}$, $p_{n}^{Y*}$, and $p_{r}^{Y*}$, we can easily obtain Proposition 1. Thus, the proof process is omitted. □

**Proof of Proposition** **2.** According to the expressions of $q_{n}^{Y*}$, $q_{r}^{Y*}$, $p_{n}^{Y*}$, and $p_{r}^{Y*}$, we can obtain the following results: if $\beta >\frac{c_{r}}{c_{n}}$, then $\frac{\partial q_{n}^{Y*}}{\partial r_{p}}=\frac{\beta (\beta c_{n}-c_{r})}{2(\beta^{2}-2\delta \beta +\delta )}>0$; otherwise, $\frac{\partial q_{n}^{Y*}}{\partial r_{p}}<0$; if $\beta <\frac{c_{r}}{c_{n}}$, then $\frac{\partial q_{r}^{Y*}}{\partial r_{p}}=\frac{c_{r}-\beta c_{n}}{2(\beta^{2}-2\delta \beta +\delta )}>0$; otherwise, $\frac{\partial q_{r}^{Y*}}{\partial r_{p}}<0$; if $\frac{c_{r}}{c_{n}}<\beta <\delta$, then $\frac{\partial p_{n}^{Y*}}{\partial r_{p}}=\frac{\left(\delta -\beta )(\beta c_{n}-c_{r}\right)}{2e_{n}(\beta^{2}-2\delta \beta +\delta )}>0$; if $\beta <\frac{c_{r}}{c_{n}}$ or β>δ, then $\frac{\partial p_{n}^{Y*}}{\partial r_{p}}<0$; if $\beta >\frac{c_{r}}{c_{n}}$, then $\frac{\partial p_{r}^{Y*}}{\partial r_{p}}=\frac{\delta (1-\beta )(\beta c_{n}-c_{r})}{2(\beta^{2}-2\delta \beta +\delta )}>0$; otherwise, $\frac{\partial p_{r}^{Y*}}{\partial r_{p}}<0$. Therefore, Proposition 2 is proven. □

**Proof of Proposition** **3.** According to the expression of $\pi_{m}^{Y*}$, we have $\frac{\partial \pi_{m}^{Y*}}{\partial \hat{E}}=\frac{\beta e_{n}\Delta_{r}-2\delta \hat{E}}{\beta^{2}e_{n}^{2}}$. Since $\hat{E}<\frac{\beta e_{n}(\Delta_{r}-\beta \Delta_{n})}{2\delta (1-\beta )}={\hat{E}}_{2}$ and ${\hat{E}}_{1}-{\hat{E}}_{2}=\frac{\beta^{2}e_{n}(\Delta_{n}-\Delta_{r})}{2\delta (1-\beta )}>0$ (i.e.,${\hat{E}}_{1}>{\hat{E}}_{2}$), there always exists $\hat{E}<\frac{\beta e_{n}\Delta_{r}}{2\delta }={\hat{E}}_{1}$, namely, $\frac{\partial \pi_{m}^{Y*}}{\partial \hat{E}}>0$. Similarly, we can also obtain $\frac{\partial \pi_{m}^{Y*}}{\partial \hat{E}}>0$ in the other two conditions. Moreover, $\frac{\partial \pi_{m}^{Y*}}{\partial r_{p}}=\frac{2[\delta (c_{n}-c_{r})-\beta (\delta c_{n}-c_{r})]\hat{E}+e_{n}(\delta -\beta )(c_{r}-\beta c_{n})-e_{n}{\left(c_{r}-\beta c_{n}\right)}^{2}(1+r_{0}-r_{p})}{2e_{n}(\beta^{2}-2\delta \beta +\delta )}$; thus, if $r_{p}>r_{p}^{m}=\frac{e_{n}{\left(c_{r}-\beta c_{n}\right)}^{2}(1+r_{0})+e_{n}(\beta -\delta )(c_{r}-\beta c_{n})-2[\delta (c_{n}-c_{r})-\beta (\delta c_{n}-c_{r})]\hat{E}}{e_{n}{\left(c_{r}-\beta c_{n}\right)}^{2}}$, then $\frac{\partial \pi_{m}^{Y*}}{\partial r_{p}}>0$; otherwise, $\frac{\partial \pi_{m}^{Y*}}{\partial r_{p}}<0$. Therefore, Proposition 3 is proven. □

**Proof of Proposition** **4.** According to the expression of $\pi_{c}$, we have $\frac{\partial \pi_{c}}{\partial r_{p}}=\frac{\left(\delta -\beta )(c_{r}-\beta c_{n})-{\left(c_{r}-\beta c_{n}\right)}^{2}(1+r_{0}-r_{p}\right)}{4(\beta^{2}-2\delta \beta +\delta )}$; thus, if $r_{p}>r_{p}^{c}=\frac{\left(c_{r}-\beta c_{n})(1+r_{0})+(\beta -\delta \right)}{c_{r}-\beta c_{n}}$, then $\frac{\partial \pi_{c}}{\partial r_{p}}>0$; otherwise, $\frac{\partial \pi_{c}}{\partial r_{p}}<0$. Therefore, Proposition 4 is proven. □

**Proof of Lemma** **3.** According to the expression of $\pi_{b}$, we have $\frac{\partial \pi_{b}}{\partial r_{p}}=\frac{2e_{n}{\left(c_{r}-\beta c_{n}\right)}^{2}(r_{0}-r_{p})+e_{n}{\left(c_{r}-\beta c_{n}\right)}^{2}+e_{n}(c_{r}-\beta c_{n})(\beta -\delta )-2\hat{E}[\delta (c_{n}-c_{r})-\beta (\delta c_{n}-c_{r})]}{2e_{n}(\beta^{2}-2\delta \beta +\delta )}$. Since $\frac{\partial \pi_{b}}{\partial r_{p}^{2}}=\frac{-{\left(c_{r}-\beta c_{n}\right)}^{2}}{\beta^{2}-2\delta \beta +\delta }<0$, this problem has a maximum point. Then, we let $\frac{\partial \pi_{b}}{\partial r_{p}}=0$ and $0<r_{p}^{*}<r_{0}$; thus, Lemma 3 is proven. □

**Proof of Proposition** **5.** According to the expression of $r_{p}^{*}$, we have $\frac{\partial r_{p}^{*}}{\partial \delta }=\frac{2\hat{E}[\beta c_{n}-(c_{n}-c_{r})]+e_{n}(\beta c_{n}-c_{r})}{2e_{n}{\left(c_{r}-\beta c_{n}\right)}^{2}}$. Then, we can obtain $\frac{\partial r_{p}^{*}}{\partial \delta }>0\Rightarrow \beta >\frac{2\hat{E}(c_{n}-c_{r})+e_{n}c_{r}}{(2\hat{E}+e_{n})c_{n}}$. Therefore, Proposition 5 is proven. □

**Proof of Proposition** **6.** According to the expression of $r_{p}^{*}$, we have $\frac{\partial r_{p}^{*}}{\partial \delta }=\frac{2\hat{E}(\delta c_{n}-c_{r})(\beta c_{n}+c_{r})-4\hat{E}\cdot \delta c_{n}(c_{n}-c_{r})-e_{n}(c_{r}-\beta c_{n})(\delta c_{n}-c_{r})}{2e_{n}{\left(c_{r}-\beta c_{n}\right)}^{3}}$. In addition, we also have $\beta >\frac{4\hat{E}\cdot \delta c_{n}(c_{n}-c_{r})-2\hat{E}\cdot c_{r}(c_{n}-c_{r})+e_{n}c_{r}(\delta c_{n}-c_{r})}{\left(2\hat{E}+e_{n})c_{n}(\delta c_{n}-c_{r}\right)}\Rightarrow 2\hat{E}(\delta c_{n}-c_{r})(\beta c_{n}+c_{r})>4\hat{E}\cdot \delta c_{n}(c_{n}-c_{r})+e_{n}(c_{r}-\beta c_{n})(\delta c_{n}-c_{r})$ and $\beta <\frac{c_{r}}{c_{n}}\Rightarrow {\left(c_{r}-\beta c_{n}\right)}^{3}>0$. Since the inequality $\frac{4\hat{E}\cdot \delta c_{n}(c_{n}-c_{r})-2\hat{E}\cdot c_{r}(c_{n}-c_{r})+e_{n}c_{r}(\delta c_{n}-c_{r})}{\left(2\hat{E}+e_{n})c_{n}(\delta c_{n}-c_{r}\right)}>\frac{c_{r}}{c_{n}}$ is always tenable, Proposition 6 is proven. □

**Proof of Proposition** **7.** According to the expression of $r_{p}^{*}$, we have $\frac{\partial r_{p}^{*}}{\partial \hat{E}}=-\frac{\hat{E}[\delta (c_{n}-c_{r})-\beta (\delta c_{n}-c_{r})]}{e_{n}{\left(c_{r}-\beta c_{n}\right)}^{2}}<0$. Additionally, substituting $q_{n}^{Y*}$, $q_{r}^{Y*}$, and $r_{p}^{*}$ into $\pi_{b}$, we have $\pi_{b}^{*}=\frac{\begin{array}{l} 2\hat{E}[\delta (c_{n}-c_{r})-\beta (\delta c_{n}-c_{r})](r_{0}-r_{p}^{*})+e_{n}(c_{r}-\beta c_{n})(\delta -\beta )(r_{0}-r_{p}^{*})\\ -e_{n}{\left(c_{r}-\beta c_{n}\right)}^{2}(r_{0}-r_{p}^{*})-e_{n}{\left(c_{r}-\beta c_{n}\right)}^{2}{\left(r_{0}-r_{p}^{*}\right)}^{2} \end{array}}{2e_{n}(\beta^{2}-2\delta \beta +\delta )}$. Then, we can obtain $\frac{\partial \pi_{b}^{*}}{\partial \hat{E}}=\frac{\begin{array}{l} 2(r_{0}-r_{p}^{*})\{[\delta (c_{n}-c_{r})-\beta (\delta c_{n}-c_{r})]+e_{n}{\left(c_{r}-\beta c_{n}\right)}^{2}\cdot \frac{\partial r_{p}^{*}}{\partial \hat{E}}\}+\\ \{e_{n}{\left(c_{r}-\beta c_{n}\right)}^{2}-e_{n}(c_{r}-\beta c_{n})(\delta -\beta )-2\hat{E}[\delta (c_{n}-c_{r})-\beta (\delta c_{n}-c_{r})]\}\cdot \frac{\partial r_{p}^{*}}{\partial \hat{E}} \end{array}}{2e_{n}(\beta^{2}-2\delta \beta +\delta )}$. Let $H=[\delta (c_{n}-c_{r})-\beta (\delta c_{n}-c_{r})]>0$, so that we have $\frac{\partial \pi_{b}^{*}}{\partial \hat{E}}=\frac{H}{e_{n}(\beta^{2}-2\delta \beta +\delta )}\cdot \frac{2H\hat{E}-e_{n}{\left(c_{r}-\beta c_{n}\right)}^{2}-e_{n}(c_{r}-\beta c_{n})(\beta -\delta )}{2e_{n}{\left(c_{r}-\beta c_{n}\right)}^{2}}=\frac{H(r_{0}-r_{p}^{*})}{e_{n}(\beta^{2}-2\delta \beta +\delta )}>0$. Therefore, Proposition 7 is proven. □
